# Editorial: Insights in oral cancers: 2023

**DOI:** 10.3389/froh.2024.1507867

**Published:** 2024-10-28

**Authors:** Ricardo D. Coletta, Ali-Farid Safi, Arnab Pal, Rogelio González-González

**Affiliations:** ^1^ Department of Oral Diagnosis and Graduate Program in Oral Biology, School of Dentistry, University of Campinas, Piracicaba, Brazil; ^2^ Faculty of Medicine, Center for Craniomaxillofacial Surgery, University of Berne, and Craniologicum, Berne, Switzerland; ^3^ Department of Biochemistry, Postgraduate Institute of Medical Education and Research, Chandigarh, India; ^4^ Research Department, School of Dentistry, Juarez University of the State of Durango, Durango, Mexico

**Keywords:** oral cancer, diagnosis, treatment, prognosis, cancer stem cell, oral potentially malignant disorders (OPMD), systematic review

**Editorial on the Research Topic**
Insights in oral cancers: 2023

## Introduction

Oral cancer, represented in the vast majority of the cases by the oral squamous cell carcinoma (OSCC), is the most common and lethal malignant neoplasm in the head and neck region (1). It is a major public health problem imposing important socio-economic issues, mainly in countries with low and very low human development indexes, and its burden is increasing (2). The efforts to better understand the many aspects related to OSCC clinical presentation (including the oral potentially malignant disorders (OPMD) that may precede OSCC development), histopathological features and genetic and molecular biology have notably changed its diagnosis, treatment and prognosis, but grand challenges persist (3). This Research Topic was set to receive articles bringing insights on current challenges of oral cancers. It received 10 articles that explore several features of the oral cancers, from early-onset as a OPMD to cellular and molecular drivers and prognosis biomarkers, highlighting clinical and pathological implications for oral cancer progression, personalized therapy and patient's outcomes.

Oral leukoplakia (OL) is the most prevalent OPMD, with a malignant transformation proportion ranging from 1.1% to 40.8% (4). Although clinical and pathological (presence of epithelial dysplasia) features have been related to malignant transformation (5, 6), it is still difficult to forecast which OL will progress to oral cancer. Based on a systematic review and meta-analysis, Normando et al. performed an in-depth analysis of various potential protein biomarkers for malignant transformation of OL. From a total of 173 distinct proteins, 18 proteins were subjected to quantitative assessment with meta-analysis, and 8 of them, including pRb, cadherin-1, mdm2, PD-L1, mucin-4, periostin and cytokeratins 13 and 19, showed statistically different levels between OL and OSCC, suggesting that may be related to increased risk of OL malignant transformation. Anaya-Saavedra and Vázquez-Garduño review the many faces of the oral human papillomavirus (HPV)-associated dysplasia, from clinical and pathological features to the prognosis and progression risk. Although described originally in 1986, only in recent years it has gained more attention due to the HPV role in oral carcinogenesis. The authors emphasize the importance of the accurate diagnosis, particularly among high-risk patients, to facilitate early intervention and offer a critical advantage in its management.

The complex crosstalk between tumor cells and the components of the microenvironment may carry out both pro- and anti-tumor activities in both early and advanced stages of the oral cancer (7). González-Arriagada et al. demonstrated that the microenvironment, containing factors produced by fibroblasts and immune-inflammatory cells and components of the extracellular matrix, provides an active and particular milieu that may facilitate the progression of OL to OSCC. Moreover, the authors discussed the promising results of a clinical trial with anti-PD-1 immunotherapy (nivolumab) on treatment of OL with high-grade epithelial dysplasia. The crucial role played by tumor microenvironment (TME) in OSCC immunomodulation was assessed also by Xavier et al. In their review, the authors provided several evidences of role of cancer-stem cells (CSC) and other stromal cells of the TME on promotion of immunosuppression and reduced anti-tumor activity of natural killer (NK) and T cells in response to CSC-stimuli, and pointed towards the necessity of multimodality therapies that disrupt the negative impact of CSC and TME on activation of NK and T cells against the tumor cells.

OSCC complexity and heterogeneity play critical impacts in diagnosis, treatment response, therapeutic failure and prognosis (8). Three articles of this Research Topic explored potential diagnostic and prognostic markers for OSCC. The first article was a systematic review on nodal tumor volume (NTV) and OSCC prognosis (Bernasconi et al.). After adopting the PRISMA (Preferred Reporting Items for Systematic Reviews and Meta-analyses) guidelines, the authors proposed that larger NTV could be used as a negative prognostic marker for OSCC, offering additional prognostic information than N category alone. However, only three studies were included in the systematic review, precluding any recommendation for clinical application, but guaranteeing further investigation. The second article, a retrospective study with 165 patients with OSCC, analyzed the prognostic impact of sarcopenia in OSCC (Takayama et al.). Using computed tomography (CT) images, sarcopenia was assessed by two methods, single muscle or whole muscle segmentation. Both methods provide valuable prognostic information for OSCC patients, with slight advantage of single muscle over whole muscle assessment. The third article explored the recent advances in minimally invasive biomarkers of OSCC (Suri et al.). Despite of promising results, particularly those from blood, saliva, buccal swabs and other body fluids of the patients (liquid biopsy) with the detection of cell free DNA (cfDNA), non-coding RNAs, some proteins and circulating tumor cells, none is approved as a biomarker for oral cancer patients in clinics. The authors reinforce the importance of validation in large and independent cohorts, which will definitely allow clinical applications.

The review by Antonelli et al. aimed to present a comprehensive overview of the current understanding of how ferroptosis, a non-apoptotic programmed cell death depending on iron and lipid peroxide accumulation, contributes to OSCC. The authors reported the regulatory mechanisms of ferroptosis in OSCC, its association with tumor immunity and prognosis, and discussed the perspective of inducing ferroptosis as a novel strategy to directly treat OSCC or, alternatively, to improve sensitivity to other approaches. Mozaffari et al. examined the expression of glucocorticoid-inducible proteins in HPV-positive oropharyngeal squamous cell carcinoma (which is closest related to OSCC), and found differential patterns of localization and expression of GILZ, Annexin-A1, SGK-1 and pSGK-1, suggesting that the glucocorticoid system may contribution to HPV pathogenesis in oropharyngeal squamous cell carcinomas. Systematic reviews have become a very important tool to make evidence-based conclusions, after gathering information from multiple independent studies. However, there are different types of systematic reviews, which should be chosen after a clear definition of the research question. The article published by Guerra et al. aimed to exemplify the different types of systematic reviews frequently used in OSCC, highlighting the importance of well-formulated research questions and of the multiple steps to produce high-quality conclusions.

This Research Topic provides a series of excellent articles that delve into various aspects of OSCC, providing the foundation for more effective strategies to improve diagnosis, treatment and prognosis of patients with OSCC. The collective insights from these articles highlight the potential for personalized therapies and early intervention. Continued studies with clear planning, strategies and protocols are essential to overcome the challenges posed by OSCC.
